# Efficacy of transcranial direct current stimulation on cognitive function in patients with Parkinson's disease: a systematic review and meta-analysis

**DOI:** 10.3389/fnagi.2025.1495492

**Published:** 2025-02-19

**Authors:** Shenhong Ma, Weisheng Zhuang, Xu Wang, Di Zhang, Heling Wang, Qiaohua Han, Qixin Ding, Yuefang Li, Wanyue Li, Tianshu Li

**Affiliations:** ^1^School of Rehabilitation Medicine, Henan University of Chinese Medicine, Zhengzhou, China; ^2^Department of Rehabilitation, Henan Provincial People's Hospital, School of Rehabilitation Medicine, Henan University of Chinese Medicine, Zhengzhou, China; ^3^School of Clinical Medicine, Henan University, Zhengzhou, China; ^4^Department of Radiology, Henan Provincial People's Hospital, Zhengzhou, China; ^5^Zhengzhou First People's Hospital, Zhengzhou, China

**Keywords:** tDCS, Parkinson's disease, cognitive function, meta-analysis, randomized controlled trial

## Abstract

**Objective:**

To assess the therapeutic effect of tDCS on cognitive function in patients with Parkinson's disease.

**Methods:**

From the start of the library's construction until June 24, 2024, we searched the following databases for literature: PubMed, Embase, Web of Science, Cochrane Library, China National Knowledge Infrastructure (CNKI), Wanfang, China Science and Technology Journal Database (VIP), and China Biomedical Literature Database (CBM). We also looked through the references in the articles. The improvement of overall cognition in patients with Parkinson's disease with tDCS was the primary outcome indicator. The improvement of executive function, memory, attention, language, quality of life, and depression with tDCS were the secondary outcome indicators. Two researchers extracted data independently, with a third researcher mediating in the event of a dispute. The Cochrane risk of bias tool was used to evaluate the quality of the included literature.

**Results:**

A total of 23 articles encompassing 874 subjects were included. tDCS has shown significant efficacy on overall cognition (SMD = 0.73, 95% CI = 0.57 to 0.89, I^2^ = 0%, *P* < 0.00001), particularly in the areas of executive function (SMD = −0.32, 95% CI = −0.56 to −0.07, I^2^ = 0%, *P* = 0.01) and language function (SMD = 0.5, 95% CI = 0.2 to 0.8, I^2^ = 0%, P = 0.001). Furthermore, the clinical efficacy of tDCS was enhanced with a stimulation intensity of 2 mA (SMD = 0.76, 95% CI = 0.58 to 0.93, I^2^ = 7%, *P* < 0.00001), a stimulation duration of ≥25 min (SMD = 0.70, 95% CI = 0.49 to 0.91, I^2^ = 6%, *P* < 0.00001), and a minimum of 10 stimulation sessions (SMD = 0.74, 95% CI = 0.56 to 0.92, I^2^ = 0%, *P* < 0.00001). Furthermore, tDCS has shown efficacy in alleviating depressive mood (SMD = −0.46, 95% CI = −0.79 to −0.13, I^2^ = 0%, *P* = 0.006).

**Conclusion:**

tDCS demonstrated substantial efficacy in enhancing overall cognition in patients with PD. The efficacy of tDCS was obvious in executive function, language, and depressive mood. Nonetheless, a substantial quantity of rigorous clinical trials on tDCS for cognitive function in patients with PD remains necessary in the future.

## 1 Introduction

Parkinson's disease (PD) is primarily characterized by various movement disorders, including impairments in ambulation and balance. As the disease advances, patients also display non-motor symptoms, such as hyposmia, cognitive decline, and sensory abnormalities, with cognitive decline being the most prevalent non-motor symptom (Aarsland et al., 2017). Patients with PD initially show subjective cognitive decline, which subsequently advances to moderate cognitive impairment (PD-MCI) and ultimately progresses to dementia (PDD) (Aarsland et al., 2017, 2021). About 50% of people diagnosed with PD will develop cognitive impairment within 6 years of their diagnosis, and projections indicate that by 2050, there will be 12 million individuals afflicted with Parkinson's worldwide, with a prevalence rate in men ~1.4 times that of women, and this scenario will impose a significant burden on society and profoundly affect the daily lives of patients, necessitating the urgent development of effective treatments (GBD, 2018; Chandler et al., 2021). There are a variety of causes of cognitive impairment in patients with PD, including synaptic changes, neuronal inflammation, structural changes in the brain, genetic variants, and aging (Hirsch and Hunot, 2009; Lashuel et al., 2013; Emre et al., 2014; Lee et al., 2014; Hopfner et al., 2020; Aarsland et al., 2021). Currently, the major treatment techniques are medication, but the treatments have side effects such as dizziness, nausea, vomiting, and so on. Patients may also acquire a certain degree of drug resistance (Zhang et al., 2020). Other non-pharmacological treatments, such as routine cognitive training and physical activity for addressing cognitive deficiencies in patients with PD, exhibit limited empirical support and are currently in the process of clinical evaluation (Emre et al., 2014; Aarsland et al., 2021). Consequently, there are no definitive and efficacious treatments, and long-term medication is typically necessary to enhance cognitive deficits in individuals with PD.

Transcranial direct current stimulation (tDCS) is a non-invasive technique that administers weak direct current to the scalp to influence neural activity in the brain. tDCS is noninvasive, relatively safe, cost-effective, easy to administer, and well tolerated and is widely used to treat depression, Alzheimer's disease, moderate cognitive impairment, and a variety of psychiatric and neurological disorders (Meinzer et al., 2015; Teselink et al., 2021; Aust et al., 2022; Woods et al., 2016; Zhao et al., 2017). In addition, tDCS has shown great potential in enhancing cognitive functioning, especially executive functioning and memory, in patients with PD, and in the future, it may become a promising treatment for cognitive deficits in patients with PD; however, the efficacy of tDCS is greatly influenced by parameter settings, including current intensity, stimulation duration, and electrode placement, which have been subjects of considerable debate and research focus (Zhao et al., 2017; Lawrence et al., 2018; Aksu et al., 2022; Ruggiero et al., 2022). Consequently, due to the clinical significance of tDCS in addressing cognitive deficits in patients with PD, this study further clarified the effectiveness of tDCS on overall cognition and various cognitive domains in this population while also performing subgroup analyses of the stimulus parameters that affected efficacy.

## 2 Methods

A comprehensive review and meta-analysis of published studies was conducted without the need for patient consent or ethical review (Higgins and Thompson, 2002). This study strictly adhered to the Preferred Reporting Items for Systematic Reviews and Meta-Analyses (PRISMA) guidelines (Moher et al., 2009). This systematic evaluation protocol is already registered with PROSPERO (reference number: CRD42024553573).

### 2.1 Search strategy

We conducted a search in Embase, PubMed, Web of Science, Cochrane Library, China National Knowledge Infrastructure (CNKI), Wanfang Database, China Science and Technology Journal Database (VIP), and China Biomedical Literature Database (CBM) and additionally examined references. All publications were published prior to 24 June 2024 with the search phrases “tDCS OR transcranial direct current stimulation,” “cognitive function OR cognitive,” and “Parkinson Disease.” Inclusion criteria were the following: (1) Participants were individuals diagnosed with PD; (2) tDCS was employed as the intervention; (3) at least one outcome measure evaluated cognitive function; and (4) the study was a RCT. Exclusion criteria were the following: (1) The subjects were not diagnosed with PD; (2) there was no tDCS intervention; (3) data were unavailable; and (4) the complete text could not be found.

### 2.2 Data extraction and outcome measures

We gathered information and data encompassing authors, publication year, region, disease type, disease duration, age, sex ratio, subject count, interventions in experimental and control groups, combined treatments, stimulation intensity, electrode locations, stimulation sessions, and each outcome measure. Data extraction was conducted individually by both researchers (WZ and SM), and disputes were resolved by a third researcher (XW) when they arose. In the absence of raw data, we utilized the Java tool GetData Graph Digitizer 2.26 to obtain the data from graphs. Primary outcome metrics were the efficacy of tDCS on overall cognition in individuals with PD, and the efficacy on executive function, memory, attention, language, depressed mood, and quality of life were used as the secondary outcome indicators.

### 2.3 Quality assessment

An independent assessment of the quality of the included literature was made by two investigators, with a third researcher intervening solely in cases of unresolved ambiguities. The Cochrane Risk of Bias Tool was adopted to evaluate literature collected for risk of bias, encompassing selecting bias, implementing bias, measuring bias, and following up bias.

### 2.4 Statistical analysis

Data were analyzed with Stata 12.0 and Review Manager version 5.4. The standardized mean difference (SMD) and 95% confidence interval (CI) were utilized to represent the final combined effect for continuous outcome indicators with varying scales, while the mean difference (MD) and 95% CI were employed for outcome indicators with identical scales (Murad et al., 2019). A fixed model was employed when the heterogeneity of the outcome indicators was below 50%, and a random-effects model and sensitivity analysis were utilized when the heterogeneity was 50% or above. Ultimately, we employed a funnel plot together with Egger's test to evaluate publication bias for the primary outcome indicators and evaluated the degree of evidence for each outcome indicator utilizing GRADE.

## 3 Results

### 3.1 Search results, study characteristics, and quality assessment

**Figure 1:**
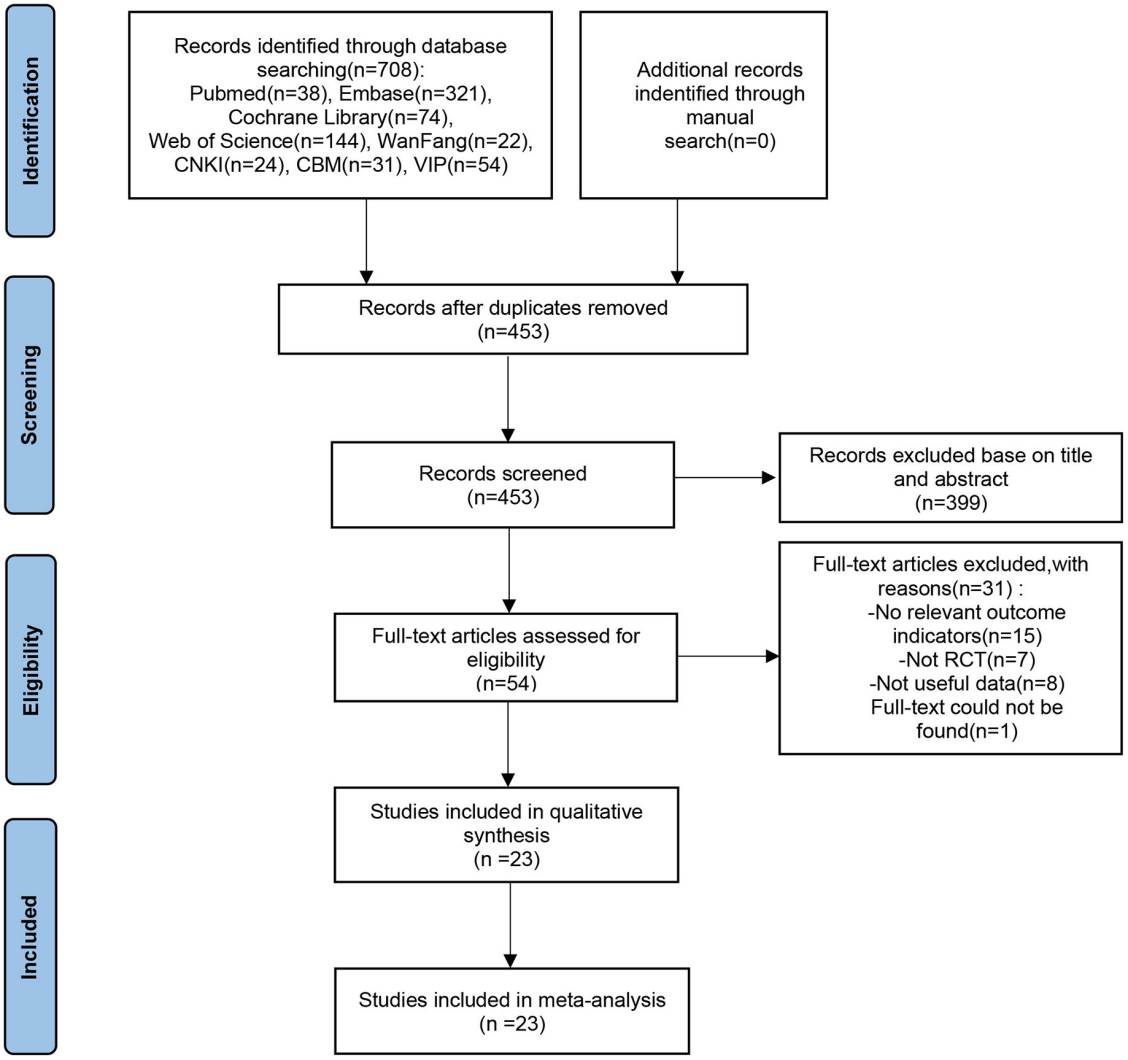
Flowchart for the literature retrieval and screening process.

**Figure 2:**
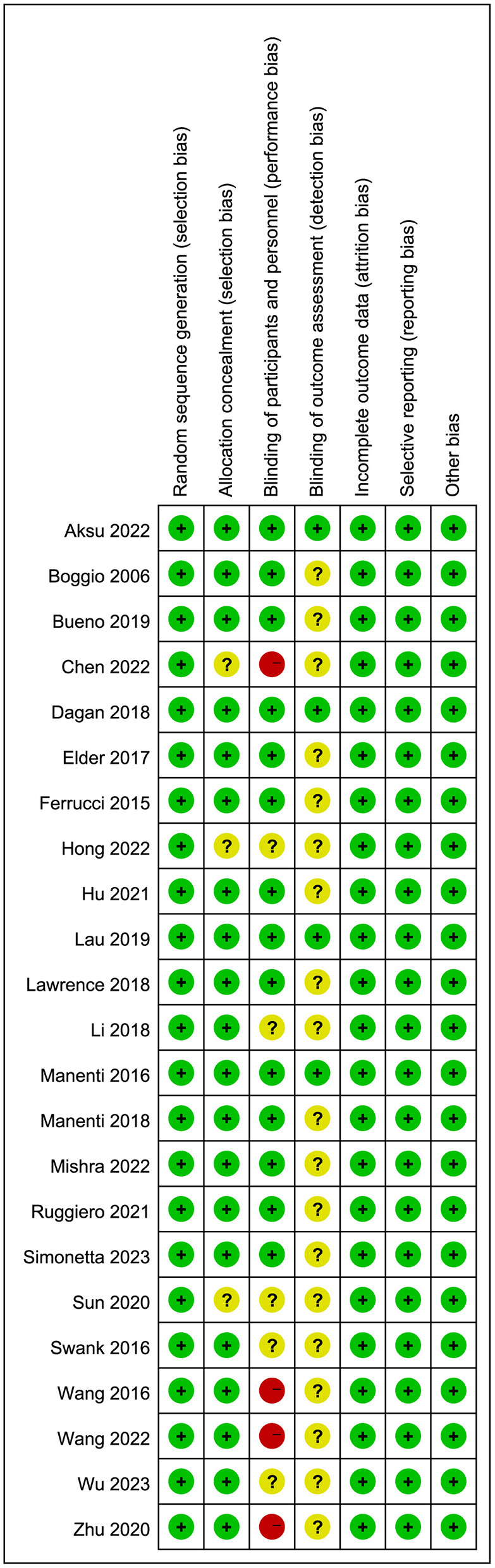
Risk of bias summary of the included RCTs.

Figure 1 illustrates the literature search and screening strategy for the current study. 708 publications were obtained after an exhaustive search, and 23 RCTs were ultimately included, encompassing 874 patients with PD, comprising 360 females and 514 males. In the experimental group, the intervention was tDCS, while the control group received sham stimulation or non-tDCS. Additional detailed information, including combined treatment modality, stimulation site, treatment duration, evaluation time, and other fundamental data, is presented in Table 1. Chen et al. (2022), Wang et al. (2016), Wang et al. (2022) and Zhu (2020) did not follow the double-blind principle during the intervention, whereas the remaining papers exhibited high quality (Figure 2).

**Table 1:** Basic information of the included studies.

**References**	**Study design**	**Region**	**Disease**	**disease duration (year)**	**Age**	**Intervention (Exp/Ctr)**	***n* (EXP/Ctr)**	**Stimulus parameter**	**Stimulus site**		**Evaluation time after treatment (day)**	**Task/measure (domain)**
									**A**	**C**		
Hong et al. (2022)	Parallel	China	Idiopathic PD	2.36 ± 0.79	69.25 ± 4.10	tDCS/non-tDCS	60 (30/30)	2 mA,20 min/session, 1 session/d, 5 d/week for 2 weeks	L-DLPFC	CSA	1	MoCA/points (general cognition)
Sun et al. (2020)	Parallel	China	PD	7.9 ± 3.4	63.5 ± 13.5	tDCS/sham	22(11/11)	2 mA,20 min/session, 1 session/d, 5 d/week for 4 weeks	L-DLPFC	CSA	1	MoCA/points(General cognition); D2 test of attention/points (attention)
Wang et al. (2022)	Parallel	China	PD	NA	64.19 ± 6.05	tDCS/non-tDCS	85 (43/42)	2 mA,25 min/session, 1 session/d, 5 d/week for 12 weeks	L-DLPFC	CSA	1	MoCA/points (general cognition); SAS/points (depression)
Lawrence et al. (2018)	Parallel	Australia	Idiopathic PD	5.43 ± 4.76	72.15 ± 6.08	tDCS/non-tDCS	14(7/7)	1.5 mA,20 min/session, 1 session/week for 4 weeks	L-DLPFC	CSA	1.84	MMSE/points (general cognition); stroop test (executive function); paragraph recall test (memory); PDQ-39/points (QOL)
Lau et al. (2019)	Crossover (WP:14days)	China	Idiopathic PD	7.8 ± 3.6	62.7 ± 6.6	tDCS/sham	10 (10/10)	2 mA,20 min/session, once	L-DLPFC	CSA	1	VWMk/d′(Memory); Go/No Go/RT (executive function)
Manenti et al. (2016)	Parallel	Italy	Idiopathic PD	7.45 ± 3.82	69.05 ± 7.35	tDCS/sham	20 (10/10)	2 mA, 25 min/session, 1 session/d, 5 d/week for 2 weeks	L-DLPFC	CSA	1.90	PD-CRS/points (general cognition); TMT-A/seconds (attention); digit span/points (memory); TMT-B/seconds (executive function); SF/points (language); BDI-II/points (depression); PDQ-39/points (QOL)
Manenti et al. (2018)	Parallel	Italy	PD	6.9 ± 3.64	64.65 ± 6.65	tDCS/sham	22 (11/11)	2 mA,25 min/session, 1 session/d, 5 d/week for 2 weeks	L-DLPFC	CSA	1.90	PD-CRS/points (general cognition); TMT-A/seconds (attention); RAVLT, immediate recall/points (memory); Go/No Go/RT (executive function); verbal fluency/points (language); BDI-II/points (depression); PDQ-39/points (QOL)
Swank et al. (2016)	Crossover (WP:7days)	USA	Idiopathic PD	7.9 ± 7.1	68.7 ± 10.2	tDCS/sham	10 (10/10)	2 mA,20 min/session, once	L-DLPFC	R- DLPFC	1	TUG cognitive/accuracy (general cognition); PDQ-39/percentage (QOL)
Ferrucci et al. (2016)	Repeated measures	Italy	Idiopathic PD	10.67 ± 3.16	74.33 ± 7.89	tDCS/sham	9(9/9)	2 mA, 25 min/session, 1 session/d for 5 d	M1	right deltoid	1	word recall/points (memory); BDI/points (depression)
Dagan et al. (2018)	Crossover (WP:2days)	USA	Idiopathic PD	9.0 ± 5.7	68.8 ± 6.8	tDCS/sham	20(20/20)	1.5 mA, 20 min/session, once	M1 and L-DLPFC	NA	1	Stroop test/accuracy (executive function)
Wang et al. (2016)	Parallel	China	PD	NA	61.5 ± 2.24	tDCS/non-tDCS	60 (30/30)	1 mA,10 min/session, 1 session/d for 10 d	L-DLPFC	CSA	1	MoCA/points (general cognition)
Li et al. (2018)	Parallel	China	PD	1.24 ± 0.56	64.36 ± 5.49	tDCS/sham	56(28/28)	2 mA,20 min/session,1 session/d for 8 weeks	Parietal and M1	CSA	1	MoCA/points (general cognition)
Hu et al. (2021)	Parallel	China	PD	2.72 ± 0.96	63.96 ± 4.99	tDCS/sham	98(49/49)	2 mA,25 min/session,1 session/d for 12 weeks	DLPFC	CSA	1	MoCA/points (general cognition)
Chen et al. (2022)	Parallel	China	PD	2.67 ± 0.35	62.32 ± 3.15	tDCS/non-tDCS	126(63/63)	2 mA,25 min/session, once	DLPFC	CSA	1	MoCA/points (general cognition)
Wu et al. (2023)	Parallel	China	PD	6.76 ± 2.91	59.4 ± 7.06	tDCS/non-tDCS	60(30/30)	2 mA,20 min/session, 1 session/d,5 d/week for 4 weeks	L-DLPFC	CSA	1	MoCA/points (general cognition)
Zhu (2020)	Parallel	China	PD	3.79 ± 2.15	77.09 ± 3.22	tDCS/non-tDCS	70(35/35)	2mA,20 min/session, 2~3 session/week for 8 weeks	L-DLPFC	CSA	1	MoCA/points (general cognition)
Aksu et al. (2022)	Parallel	Italy	idiopathic PD	4.81 ± 3.48	65.52 ± 7.49	tDCS/sham	26(13/13)	2 mA, 20 min/session, once	L-DLPFC	R- DLPFC	1.30	TMT-A/seconds (attention); WMS IR/points (memory); Stroop test interference time/seconds (executive function); SF/points (language)
Bueno et al. (2019)	Crossover (WP:7days)	UK	Idiopathic PD	NA	64.45 ± 8.98	tDCS/sham	20(20/20)	2 mA, 20 min/session, once	L-DLPFC	Right orbital frontal cortex	1	TMT-A/seconds (attention); TMT-B, seconds/(executive function); verbal fluency/number (language)
Mishra and Thrasher (2022)	Crossover (WP:7days)	USA	Idiopathic PD	4.8 ± 3.8	67.8 ± 8.3	tDCS/sham	20 (20/20)	2 mA, 30 min/session, once	L-DLPFC	CSA	1	Dual task/accuracy (attention)
Elder et al. (2017)	Crossover (WP:1day)	UK	PDD	7.39 ± 2.85	66.63 ± 8.39	tDCS/sham	38 (38/38)	2.8 mA, 20 min/session, once	L-DLPFC	Right deltoid	1	CRT/ms (attention)
Simonetta et al. (2023)	Crossover (WP:90days)	Italy	Idiopathic PD	7.9 ± 3.57	52.3 ± 4.24	tDCS/sham	10 (10/10)	2 mA,20 min/session, 1 session/d for 10 d	L-M1	CSA	1	PD-CRS/points (general cognition); PDQ-39/points (QOL)
Boggio et al. (2006)	Repeated measures	USA	Idiopathic PD	12.7 ± 8.1	61.0 ± 12.1	tDCS/sham	9 (9/9)	2 mA, 20 min/session, once	L-DLPFC	CSA	1	Three-back/accuracy (memory)
Ruggiero et al. (2022)	Crossover (WP:30days)	Italy	Idiopathic PD	13.14 ± 5.9	64.56 ± 10.27	tDCS/sham	9 (9/9)	2 mA, 20 min/session,1 session/d for 5 d	Cerebellar	Right shoulder	1	SRT/ms (attention)

A, anode; BDI, Beck Depression Inventory; C, cathode; Ctr, control group; CSA, contralateral supraorbital; CRT, choice reaction time; DLPFC, dorsolateral prefrontal cortex; Exp, experimental group; MoCA, The Montreal Cognitive Assessment; MMSE, Minimum Mental State Examination; NA, no answer; PD-CRS, Parkinson's Disease-Cognitive Rating Scale; QOL, Quality of Life; RAVLT, Rey Auditory Verbal Learning test; RT, reaction time; SAS, Self-Rating Anxiety Scale; SF, semantic fluency; TMT, Trail Making Test; WMS, Wechsler Memory Scale; WP, washout period.

### 3.2 Efficacy of tDCS in patients with PD

#### 3.2.1 Efficacy of tDCS on general cognition in patients with PD

**Figure 3:**
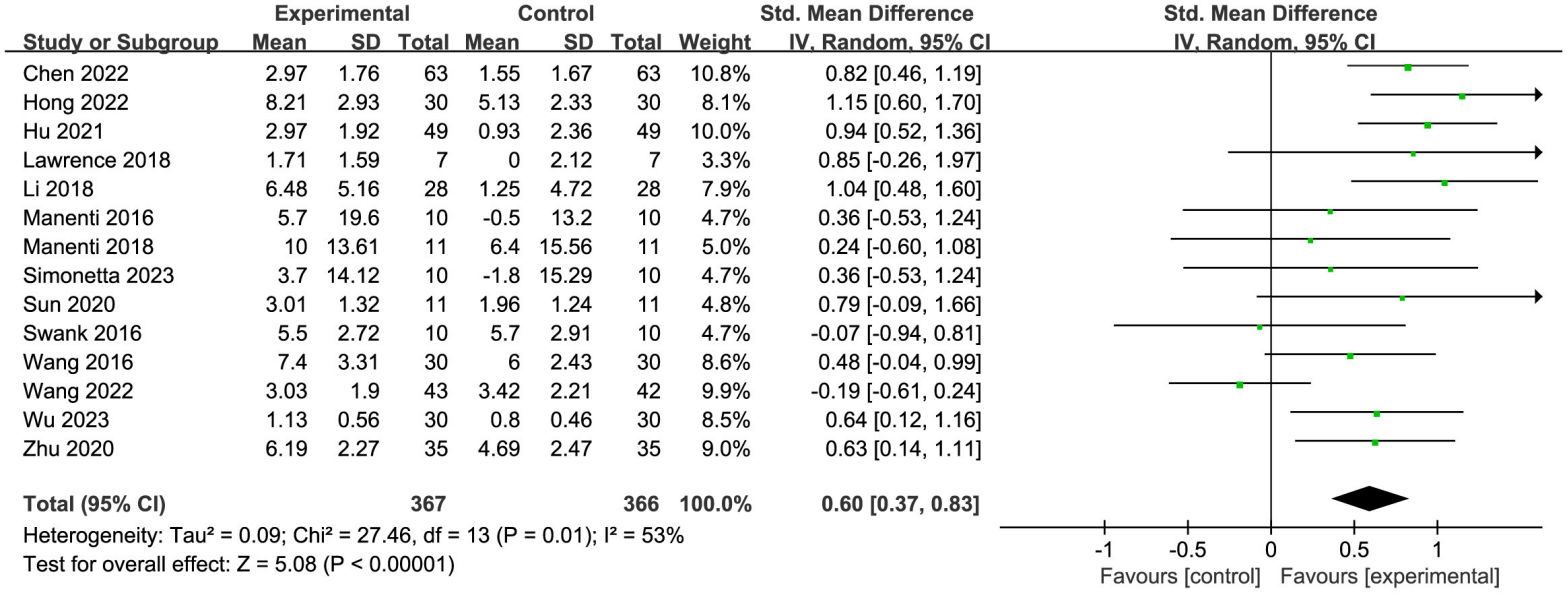
Forest plot of efficacy of tDCS on overall cognition before elimination.

**Figure 4:**
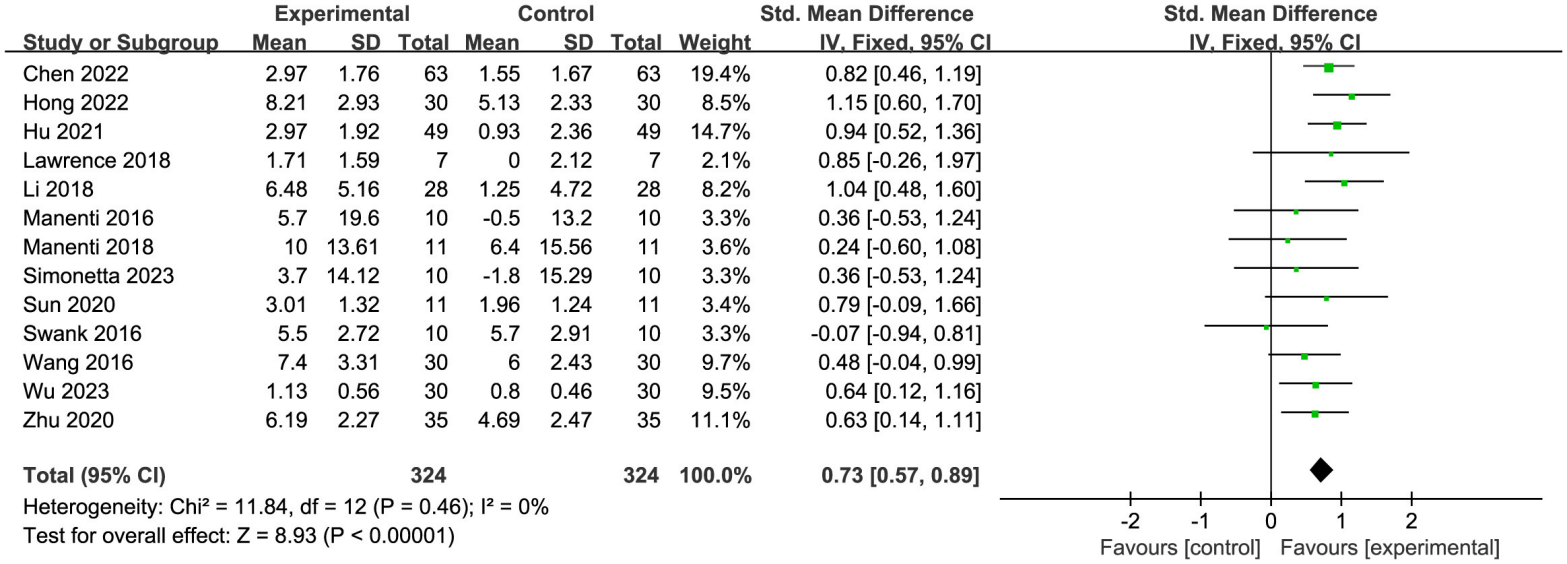
Forest plot of efficacy of tDCS on overall cognition after elimination.

Fourteen papers (Manenti et al., 2016; Swank et al., 2016; Wang et al., 2016; Lawrence et al., 2018; Li et al., 2018; Manenti et al., 2018; Sun et al., 2020; Zhu, 2020; Hu et al., 2021; Chen et al., 2022; Hong et al., 2022; Wang et al., 2022; Simonetta et al., 2023; Wu et al., 2023) assessed global cognition, and the analysis revealed a high degree of heterogeneity (Figure 3). Nonetheless, the heterogeneity diminished to 0% following the elimination of Wang et al. (2022). Consequently, we omitted this article, and it was postulated that its significant variability may be attributed to the combination treatment, stimulus intensity, or stimulus duration. The final results demonstrated substantial immediate efficacy of tDCS on overall cognition (SMD = 0.73, 95% CI = 0.57 to 0.89, I^2^ = 0%, *P* < 0.00001) (Figure 4).

**Figure 5:**
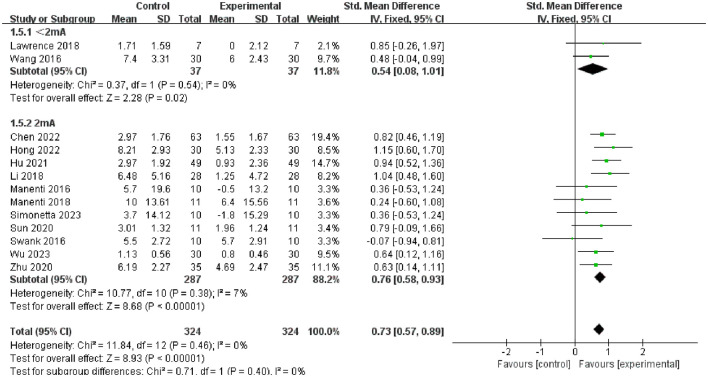
Forest plot of tDCS on overall cognition in patients with Parkinson's disease according to the subgroups of tDCS intensity.

**Figure 6:**
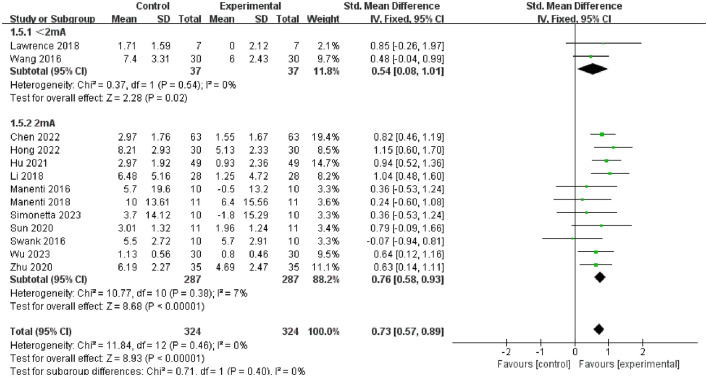
Forest plot of tDCS on overall cognition in patients with Parkinson's disease according to the subgroups of tDCS duration.

**Figure 7:**
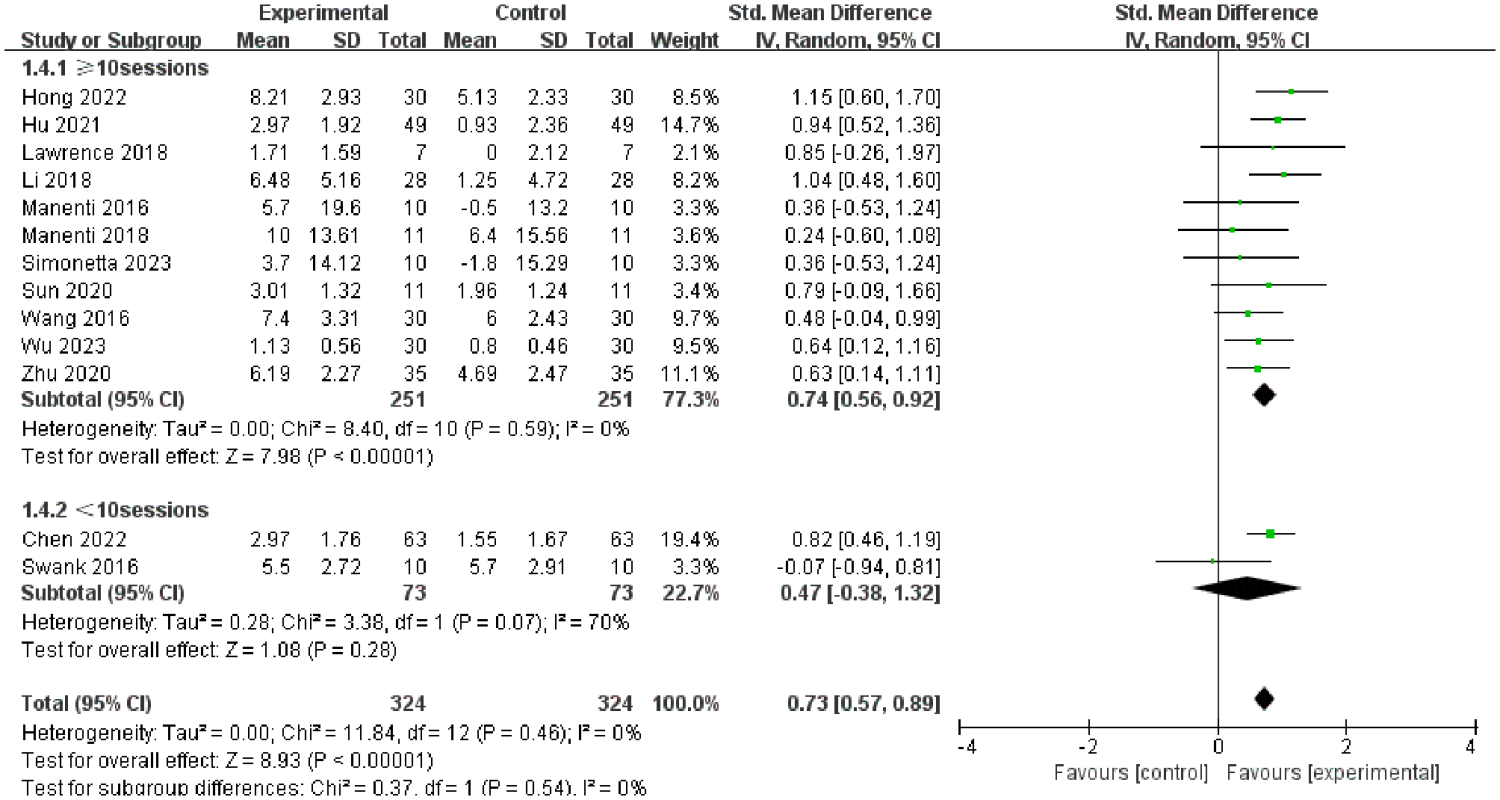
Forest plot of tDCS on overall cognition in patients with Parkinson's disease according to the subgroups of tDCS session.

Furthermore, we conducted subgroup analysis of the results to investigate factors affecting efficacy. The final results indicated that tDCS with high stimulation intensity (2 mA) (SMD = 0.76, 95% CI = 0.58 to 0.93, I^2^ = 7%, *P* < 0.00001) demonstrated greater efficacy than tDCS with low stimulation intensity (<2 mA) (SMD = 0.54, 95% CI = 0.08 to 1.01, I^2^ = 0%, P = 0.02) (Figure 5); tDCS with a longer stimulation duration (≥25 min) (SMD = 0.78, 95% CI = 0.53 to 1.03, I^2^ = 4%, P < 0.00001) was more effective than tDCS with short stimulation duration (< 25 min) (SMD = 0.70, 95% CI = 0.49 to 0.91, I^2^ = 6%, *P* < 0.00001) (Figure 6); and the efficacy of tDCS with multiple sessions (≥10 sessions) was significant (SMD = 0.74, 95% CI = 0.56 to 0.92, I^2^ = 0%, *P* < 0.00001), whereas the efficacy of tDCS with fewer sessions (< 10 sessions) was not obvious (SMD = 0.47, 95% CI = −0.38 to 1.32, I^2^ = 70%, *P* = 0.28) (Figure 7).

#### 3.2.2 Efficacy of tDCS on specific cognitive domains in patients with PD

We evaluated the effectiveness of tDCS on particular cognitive domains, namely, executive function, language, attention, and memory, in individuals with PD.

**Figure 8:**
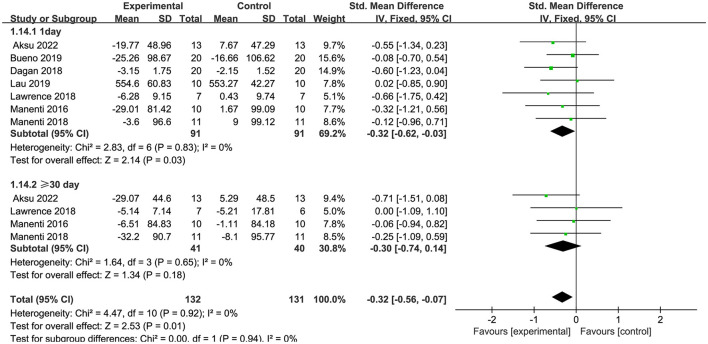
Forest plot of the efficacy of tDCS on executive function.

**Figure 9:**
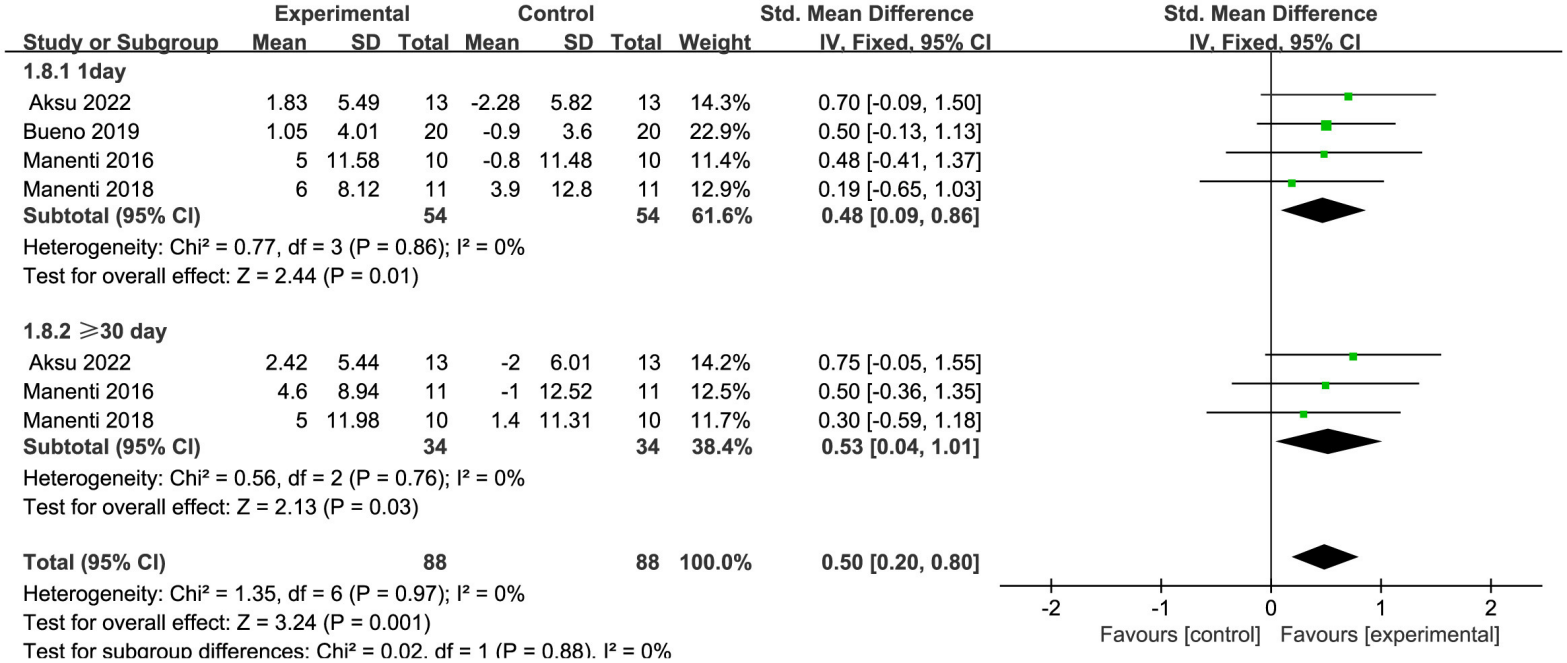
Forest plot of the efficacy of tDCS on language.

**Figure 10:**
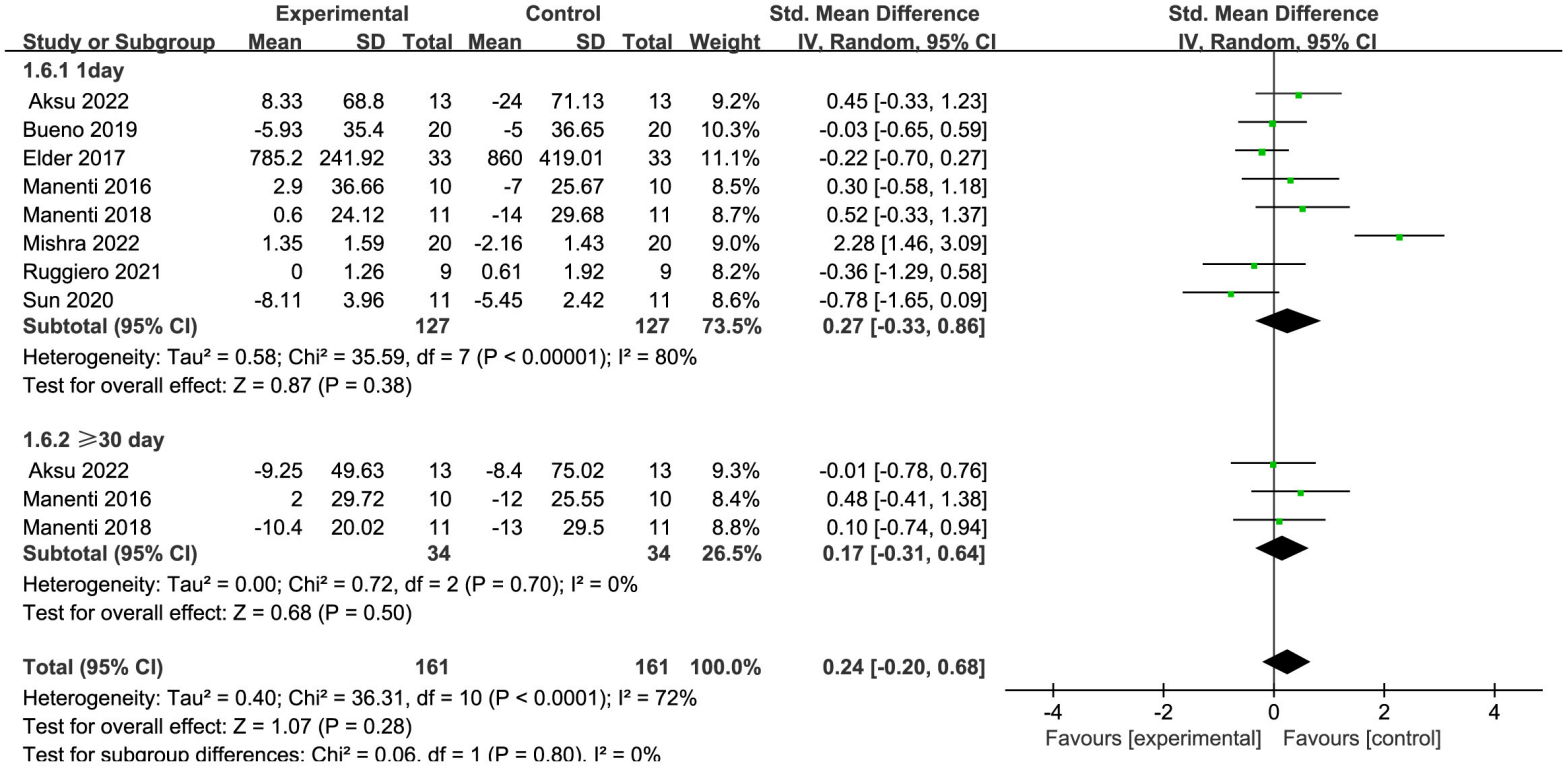
Forest plot of the efficacy of tDCS on attention.

**Figure 11:**
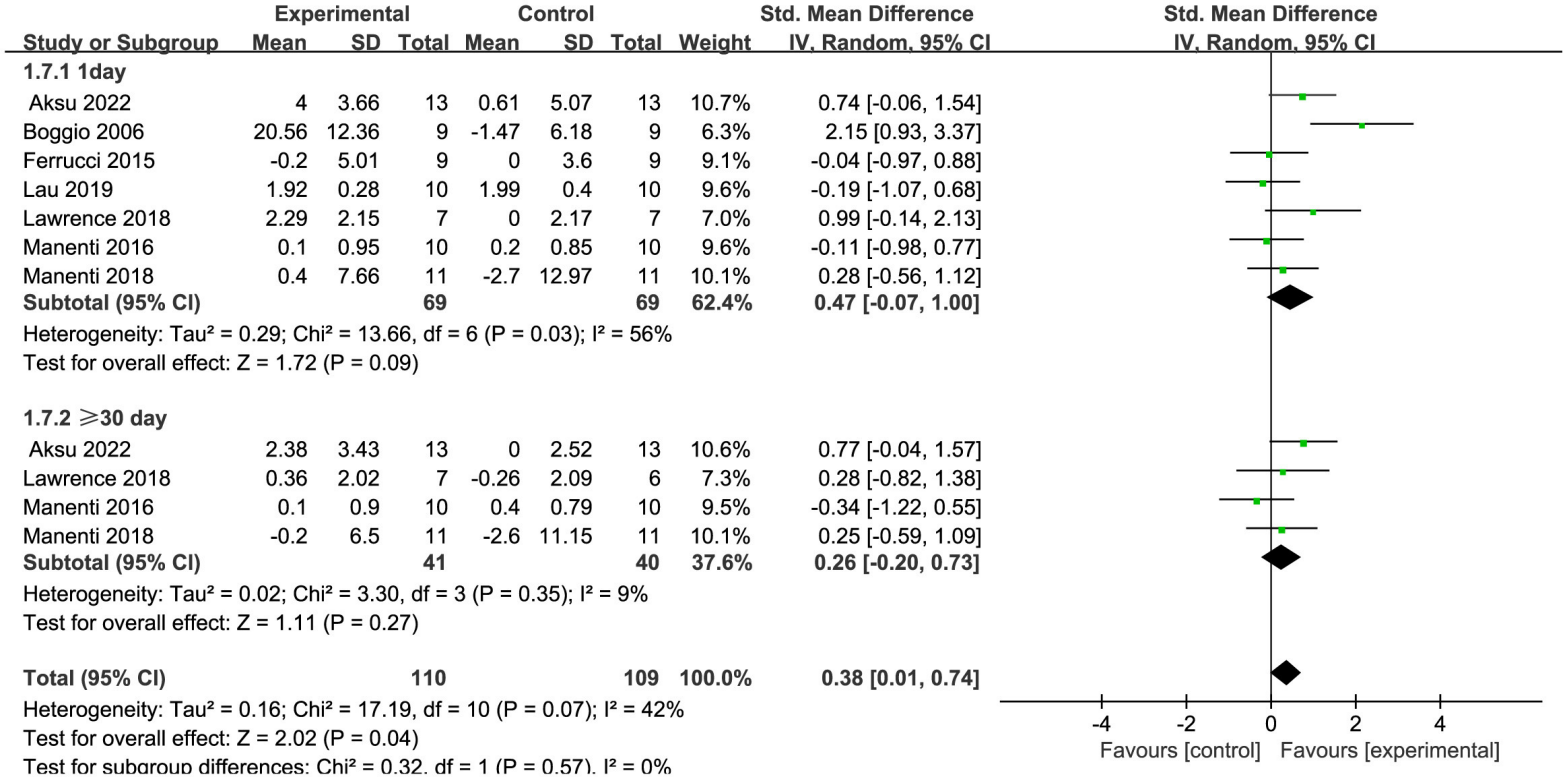
Forest plot of the efficacy of tDCS on memory.

Seven articles (Manenti et al., 2016; Dagan et al., 2018; Lawrence et al., 2018; Manenti et al., 2018; Bueno et al., 2019; Lau et al., 2019; Aksu et al., 2022) evaluated executive function and demonstrated that tDCS had a significant immediate effect on executive function in patients with PD (SMD = −0.32, 95% CI = −0.62 to −0.03, I^2^ = 0%, P = 0.03), whereas its long–term effect was insignificant (SMD = −0.30, 95% CI = −0.74 to 0.14, I^2^ = 0%, P = 0.18) (Figure 8). Four articles (Manenti et al., 2016, 2018; Bueno et al., 2019; Aksu et al., 2022) evaluated language function and demonstrated that tDCS significantly improved language function in patients with PD, exhibiting both immediate (SMD = 0.48, 95% CI = 0.09 to 0.86, I^2^ = 0%, P = 0.01) and long–term efficacy (SMD = 0.53, 95% CI = 0.04 to 1.01, I^2^ = 0%, P = 0.03) (Figure 9). Eight articles (Manenti et al., 2016; Elder et al., 2017; Manenti et al., 2018; Bueno et al., 2019; Sun et al., 2020; Aksu et al., 2022; Mishra and Thrasher, 2022; Ruggiero et al., 2022) evaluated attention and revealed no significant enhancement in attentional function among patients with PD receiving tDCS (SMD = 0.27, 95% Cl = −0.33 to 0.86, I^2^ = 80%, P=0.38) (Figure 10). Additionally, seven articles (Boggio et al., 2006; Ferrucci et al., 2016; Manenti et al., 2016; Lawrence et al., 2018; Manenti et al., 2018; Lau et al., 2019; Aksu et al., 2022) examined memory function, revealing no apparent improvements (SMD = 0.48, 95% CI = −0.05 to 1.01, I^2^ = 55%, P = 0.07) (Figure 11).

#### 3.2.3 Efficacy of tDCS on depressed mood and quality of life in patients with PD

**Figure 12:**
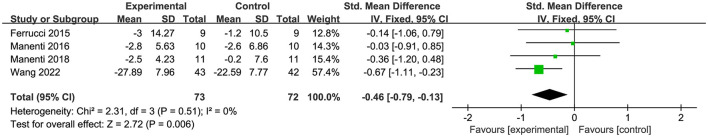
Forest plot of the efficacy of tDCS on depression.

**Figure 13:**
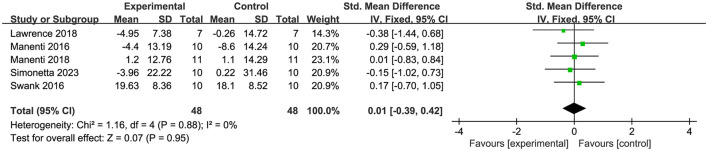
Forest plot of the efficacy of tDCS on quality of life.

Four articles (Ferrucci et al., 2016; Manenti et al., 2016, 2018; Wang et al., 2022) evaluated depressive mood, revealing that tDCS exerted a significant immediate effect on it (SMD = −0.46, 95% CI = −0.79 to −0.13, I^2^ = 0%, P = 0.006) (Figure 12). Five articles (Manenti et al., 2016; Swank et al., 2016; Lawrence et al., 2018; Manenti et al., 2018; Simonetta et al., 2023) involved the assessment of quality of life, but the results showed that tDCS had no ameliorative effect on it (SMD = 0.01, 95% CI = −0.39 to 0.42, I^2^ = 0%, P = 0.95) (Figure 13).

### 3.3 Publication bias and sensitivity analysis

**Figure 14:**
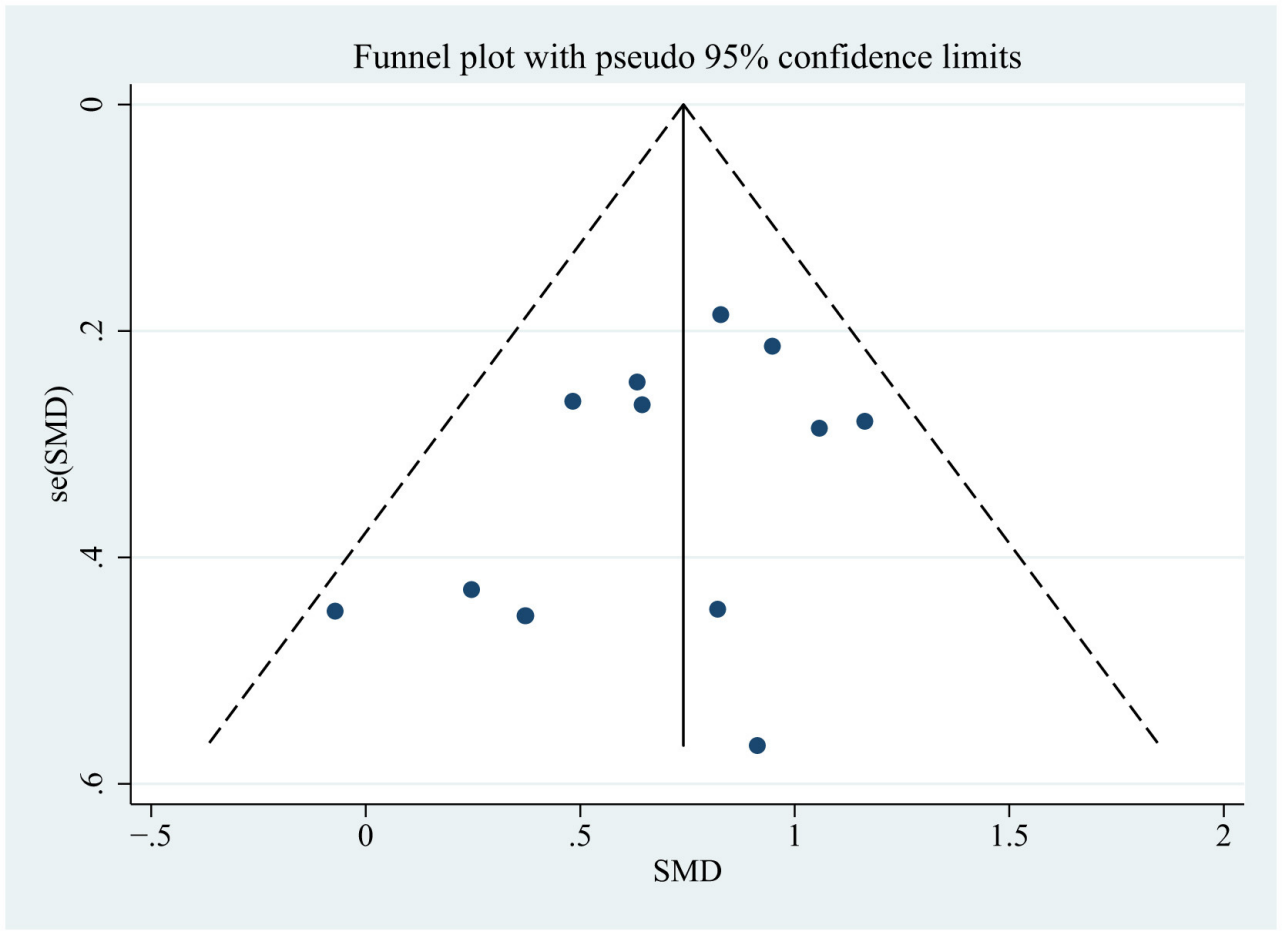
Funnel plot of the efficacy of tDCS on overall cognition.

We demonstrated that no publication bias existed for the primary outcome indicator by Egger's test (*P* = 0.112) and funnel plot (Figure 14). We performed sensitivity analyses of the primary outcomes using a piece-by-piece culling method, where a study was removed each time and a new meta-analysis was performed separately, which showed no change in the effect sizes.

### 3.4 GRADE quality evaluation results of this study

The evidence level for each outcome indicator was assessed using GRADE software, yielding the following results: (1) the quality rating for the efficacy of tDCS on overall cognition in patients with PD was high; (2) the quality rating for the efficacy of tDCS on language, executive functioning, memory, attention, quality of life, and depressive mood was moderate.

## 4 Discussion

The following is the interpretation of the results of the data analysis: (1) tDCS has a very significant efficacy in enhancing the general cognition of patients with PD, corroborating findings from previous clinical research (Lawrence et al., 2018), and indicating that tDCS may serve as a promising therapeutic intervention for cognitive deficiencies in this population. Furthermore, a stimulation intensity of 2 mA proved to be more effective, aligning with the clinical findings of Boggio et al. (2006). Additionally, the clinical efficacy of tDCS is enhanced with a stimulation duration of ≥25 min, and the stimulation sessions of ≥10 sessions are also better, thereby optimizing the stimulation parameters of tDCS; (2) tDCS demonstrates a significant enhancement in executive and language functions. Executive dysfunction is a cognitive domain that manifests early in patients with PD and significantly contributes to cognitive deficits (Muslimovic et al., 2005; Kudlicka et al., 2011); thus, the enhancement of executive function with tDCS indicates substantial potential for treating early cognitive impairments in this population; (3) tDCS improves depressive symptoms in patients with PD, suggesting it may be a primary therapy option for those experiencing depressive mood and cognitive deficits.

There are several main mechanisms by which tDCS improves cognitive deficits in patients with PD, and these therapeutic mechanisms are interconnected and interact with each other. To begin with, tDCS modulates cortical excitability and improves activity in cortical areas associated with cognitive function, thereby improving cognitive function in patients with PD. Anodic tDCS depolarizes the membrane potential of neurons in its area of action, lowering their excitatory threshold, making them more easily activated, and increasing neuronal excitability, whereas cathodic tDCS hyperpolarizes the membrane potential of neurons in its area of action, raising their excitatory threshold, making them difficult to activate, and reducing neuronal excitability (Nitsche and Paulus, 2000; Liebetanz et al., 2002). Boggio et al. demonstrated that tDCS in the left dorsolateral prefrontal cortex improved working memory function in patients with PD, and they suggested that this was due to anodic tDCS inducing neuronal depolarization in the left dorsolateral prefrontal cortex, which caused an increase in regional cortical excitability (Boggio et al., 2006). Bueno et al. (2019) similarly used anodic tDCS to increase excitability in the left dorsolateral prefrontal cortex of patients with PD, ultimately improving verbal fluency and executive function. tDCS also modulates neuroplasticity and has both immediate and long-term effects on cognitive function in patients with PD. The immediate effect is due to the fact that anodic tDCS increases neuronal excitability and neuronal firing rate, which in turn increases the efficiency of synaptic transmission (Reis et al., 2009). Doruk et al. (2014) demonstrated that tDCS has a long-term effect on executive function in patients with PD, which is consistent with the findings of the current meta-analysis. This is because anodic tDCS depolarises the neuronal membrane potential increasing the influx of Ca^2+^ and Mg^2+^, activating the N-methyl-D-aspartate receptor (NMDAR) channels, increasing the postsynaptic concentration of Ca^2+^ to promote the expression of post-synaptic densities of proteins (PSDs) and increasing the activity at glutamatergic synapses, which then promotes the formation of LTP, while cathodal tDCS promotes LTD production by reducing cortical excitability and presynaptic neurotransmitter release, in addition, LTP and LTD are the basis of neuroplasticity, LTP strengthens synaptic connectivity and LTD weakens unwanted synaptic connectivity, and together they participate in the adjustment and optimisation of neural networks, and the balance between them is very important for normal learning and memory processes (Bliss and Collingridge, 1993; Bear and Malenka, 1994; Malenka and Nicoll, 1999; Citri and Malenka, 2008; Doruk et al., 2014; Manenti et al., 2016). Moreover, neurotransmitter release may be impacted by tDCS. Research has demonstrated that patients with PD often have a loss of dopaminergic neurons and that this loss leads to over activity of the glutamatergic system, which in turn may lead to neurotoxic damage to other neurons in the brain and ultimately to cognitive dysfunction (Akcay and Tamerer, 2023). Stagg et al. (2009) found that anodic tDCS prevented the formation of γ-aminobutyric acid (GABA), while cathodic tDCS inhibited the generation of glutamate, using magnetic resonance spectroscopy (MRS). It has been shown that anodic tDCS increases excitatory neuronal activity and thus leads to a decrease in GAD-67, which is a key enzyme in the promotion of GABA synthesis, ultimately leading to a decrease in GABA concentration, while cathodic tDCS decreases neuronal activity, resulting in lower enzyme activity and ultimately lower glutamate concentrations (Levy et al., 2002; Floyer-Lea et al., 2006; Stagg et al., 2009). Nonetheless, there is still not enough clinical research that clearly shows how tDCS precisely and directly affects GABA and glutamate to enhance cognitive function in patients with PD. Further research needs to explore these mechanisms in greater depth to provide more definitive evidence. Pereira et al. (2013) found that the use of tDCS acting on the left dorsolateral prefrontal cortex significantly increased the functional connectivity of the relevant brain networks, which ultimately led to a significant improvement in verbal fluency in patients with PD, which is consistent with the present study's findings and further supports the view that tDCS can alter the cortical excitability of different regions and enhance the functional connectivity between these regions, which can lead to improved efficiency of the brain networks for information processing and ultimately improve cognitive function in patients with PD. However, further studies are required to verify and explain in detail the specific effects and action mechanisms of tDCS on cognitive function in patients with PD.

There have been published meta-analyses discussing the efficacy of tDCS on cognitive performance in individuals with PD. One of the meta-analyses concluded that tDCS seems to have a role in improving cognitive performance in individuals with PD. In this meta-analysis, the composite effect size of the overall cognitive scores indicated that tDCS did not have an effect on cognition, but one of the subgroup MoCA scores indicated that tDCS was effective in improving cognitive performance in individuals with PD (Liu et al., 2021). Two other meta-analyses also showed that tDCS did not improve cognitive function in patients with PD (Suarez-Garcia et al., 2020; Duan and Zhang, 2024). Unlike them, the network meta-analyses published by Lee et al. (2024) and Wang et al. (2024) were consistent with the results of the present study, namely that tDCS has favorable clinical effects on cognitive function in patients with PD. However, the number of studies included in these published meta-analyses is limited. This study is a larger study because of the large number of articles included. In addition, the included studies were all RCTs, so the quality of the evidence in this study was high. At the same time, we discussed the comprehensive analysis of tDCS on different cognitive domains such as attention, memory, execution, and language in patients with PD, and we also conducted a subgroup analysis of the efficacy of tDCS on the overall cognition of patients with PD, and explored the parameters that affect the efficacy, such as stimulation intensity, duration, and frequency. This study still has some limitations because most recent clinical trials have examined the short-term effectiveness of tDCS on cognitive impairment in patients with PD without evaluating the treatment's long-term effectiveness. As a result, this study primarily examined the immediate effectiveness of tDCS on improving cognitive function in patients with PD. In addition, in our meta-analysis, the varying durations of sham stimulation across studies may have influenced the magnitude of the placebo effect, potentially contributing to heterogeneity in control group outcomes. This is because longer durations of sham stimulation could enhance participants' expectations, thereby amplifying placebo responses. Future studies should aim to standardize the duration of sham stimulation to ensure consistent treatment conditions in control and experimental groups.

## 5 Conclusion

In addition to enhancing depressive symptoms, tDCS is effective in treating cognitive impairment in patients with PD, particularly in language, executive function, and general cognition. However, this study only demonstrated the immediate effect of tDCS, so more high-quality clinical studies are needed in the future to explore their long-term efficacy.

## Data Availability

The original contributions presented in the study are included in the article/supplementary material, further inquiries can be directed to the corresponding author.
